# Insular Dysfunction Reflects Altered Between-Network Connectivity and Severity of Negative Symptoms in Schizophrenia during Psychotic Remission

**DOI:** 10.3389/fnhum.2013.00216

**Published:** 2013-05-20

**Authors:** Andrei Manoliu, Valentin Riedl, Anselm Doll, Josef Georg Bäuml, Mark Mühlau, Dirk Schwerthöffer, Martin Scherr, Claus Zimmer, Hans Förstl, Josef Bäuml, Afra M. Wohlschläger, Kathrin Koch, Christian Sorg

**Affiliations:** ^1^Department of Psychiatry, Klinikum Rechts der Isar, Technische Universität MünchenMunich, Germany; ^2^Department of Neuroradiology, Klinikum Rechts der Isar, Technische Universität MünchenMunich, Germany; ^3^TUM-Neuroimaging Center, Technische Universität MünchenMunich, Germany; ^4^Department of Nuclear Medicine, Klinikum Rechts der Isar, Technische Universität MünchenMunich, Germany; ^5^Department of Neurology, Klinikum Rechts der Isar, Technische Universität MünchenMunich, Germany; ^6^Munich Center for Neurosciences Brain & Mind, Ludwig-Maximilians-Universität MünchenMunich, Germany; ^7^Department of Neurology, Christian Doppler Klinik, Paracelsus Medical University SalzburgSalzburg, Austria

**Keywords:** schizophrenia, remission, anterior insula, salience network, default mode network, central executive network

## Abstract

Schizophrenia is characterized by aberrant intrinsic functional connectivity (iFC) within and between intrinsic connectivity networks (ICNs), including the Default Mode- (DMN), Salience- (SN), and Central Executive Network (CEN). The anterior insula (AI) of the SN has been demonstrated to modulate DMN/CEN interactions. Recently, we found that the dependence of DMN/CEN interactions on SN’s right AI activity is altered in patients with schizophrenia in acute psychosis and related to psychotic symptoms, indicating a link between aberrant AI, DMN, CEN, and psychosis. However, since structural alterations of the insula are also present during psychotic remission and associated with negative symptoms, impaired AI interaction might be relevant even for psychotic remission and corresponding symptoms. Twelve patients with schizophrenia during psychotic remission (SR) and 12 healthy controls were assessed using resting-state fMRI and psychometric examination. High-model-order independent component analysis of fMRI data revealed ICNs including DMN, SN, and CEN. Scores of iFC within (intra-iFC) and between (inter-iFC) distinct subsystems of the DMN, SN, and CEN were calculated, compared between groups and correlated with the severity of symptoms. Intra-iFC was altered in patients’ SN, DMN, and CEN, including decreased intra-iFC in the left AI within the SN. Patients’ inter-iFC between SN and CEN was increased and correlated with the severity of negative symptoms. Furthermore, decreased intra-iFC of the left AI correlated with both severity of negative symptoms and increased inter-iFC between SN and CEN. Our result provides first evidence for a relationship between AI dysfunction and altered between-network interactions in schizophrenia during psychotic remission, which is related to the severity of negative symptoms. Together with our previous results, data suggest specific SN/DMN/CEN reorganization in schizophrenia with distinct insular pathways for distinct symptom dimensions.

## Introduction

Schizophrenia is a severe mental disorder associated with aberrant functional and structural connectivity within and between intrinsic connectivity networks (ICNs), including the Default Mode- (DMN), Salience- (SN), and Central Executive Network (CEN) (Menon, 2011; Palaniyappan and Liddle, 2012). ICNs are characterized by spatially consistent functional connectivity (FC) of intrinsic brain activity (Fox and Raichle, 2007; Allen et al., 2011). Since DMN, SN and CEN play a critical role in high-level cognition [and are therefore considered as core neurocognitive networks (Uddin et al., 2011)], they have been suggested to be involved in different symptom dimensions of schizophrenia (Williamson, 2007).

More specifically, the DMN includes primarily the ventromedial prefrontal cortex, the posterior cingulate cortex, bilateral inferior parietal cortex, and the middle temporal lobe and is involved in self-related/internally oriented processes (Buckner et al., 2008). The CEN includes mainly the dorsolateral prefrontal cortex and posterior parietal cortex and is involved in goal-directed/externally oriented tasks (Fox and Raichle, 2007). In schizophrenia, alterations in FC have been reported for DMN as well as CEN during both rest (Whitfield-Gabrieli et al., 2009; Rotarska-Jagiela et al., 2010; Skudlarski et al., 2010), and task (Garrity et al., 2007; Minzenberg et al., 2009; Whitfield-Gabrieli et al., 2009). Furthermore, the interaction between these networks has been reported to be disrupted in patients (Hasenkamp et al., 2011), suggesting that altered between-network interactions and thus impaired coordination of self-related processes and goal-directed tasks might underlie both positive and negative symptoms in schizophrenia (Williamson, 2007).

The SN includes primarily the anterior insular cortex and dorsal anterior cingulate cortex and is involved in detecting and orienting to salient external stimuli and internal events, including emotional, autonomic, and interoceptive informations (Seeley et al., 2007). Within the SN, the anterior insular cortex plays a crucial role in maintaining representations and updating of current and predictive salience (Singer et al., 2009; Palaniyappan and Liddle, 2012). Functional and structural alterations within the insular cortex are among the most frequently reported anomalies in schizophrenia (Palaniyappan and Liddle, 2012), including altered functional activity during tasks (Murray et al., 2008), reduced gray matter (GM) (Ellison-Wright et al., 2008), and decreased white matter (WM) fractional anisotropy (Ellison-Wright and Bullmore, 2009). Therefore, it has been suggested that functional and/or structural alterations within the insular cortex might contribute to aberrant salience processing, leading to the emergence of symptoms in schizophrenia (Palaniyappan and Liddle, 2012).

But how are anomalies in the anterior insula (AI) within the SN linked to aberrant DMN/CEN interactions in schizophrenia? Recently, it has been demonstrated that the anterior insula within the SN is crucial for modulating interactions between DMN-mediated self-related and CEN-mediated external-task directed processes in response to cognitive demands (Sridharan et al., 2008; Uddin et al., 2011). Recent models of insular dysfunction in schizophrenia hypothesized a relationship between impaired activity of the AI within the SN, disrupted DMN/CEN interaction, and different symptoms in schizophrenia (Menon, 2011; Palaniyappan and Liddle, 2012). Corresponding with these models, we demonstrated in a previous study (Manoliu et al., 2013) that the dependence of DMN/CEN interactions on SN’s right AI activity was aberrant in patients with schizophrenia during state of acute psychosis and related to psychotic symptoms. More specifically, we found that the decreased connectivity within the SN’s right AI correlated with both increased connectivity between DMN and CEN and the severity of hallucinations. These data demonstrate a specific link between right anterior insular dysfunction, aberrant inter-network connectivity, and positive symptoms in schizophrenia during psychosis. However, these data provide no information about insula’s role in psychotic remission and for negative symptoms particularly in the context of network interactions. This might be of relevance because insular alterations such as structural reorganization or aberrant reward-related activity have been demonstrated to be present during psychotic remission and to be associated with negative symptoms (Palaniyappan et al., 2011; Gradin et al., 2013). Based on these data, we suggested that insular network interactions might be aberrant also during psychotic remission and associated with negative symptoms.

To test this hypothesis, we followed the approach previously reported (Manoliu et al., 2013) and performed resting-state functional magnetic resonance imaging (rs-fMRI), which measures ongoing blood-oxygenation-level-dependent (BOLD) fluctuations, and structural magnetic resonance imaging as well as psychometric assessment in 12 patients with schizophrenia during state of psychotic remission and 12 matched healthy controls (HCs). Rs-fMRI data were decomposed by high-model-order independent component analysis (ICA) into spatially independent z-maps of functionally coherent brain areas and corresponding time courses (TCs) of component activity (Calhoun et al., 2001). From these spatial maps, we selected those representing the SN, DMN, and CEN. Main outcome measures were Pearson’s correlation between-network time series, reflecting inter-network intrinsic functional connectivity (inter-iFC), and components’ z-maps, reflecting the intra-network intrinsic functional connectivity (intra-iFC). We controlled our analyses for effects of age, sex, medication, and structural anomalies.

## Materials and Methods

### Participants

Twelve patients with schizophrenia during state of remission and 12 age and sex-matched HCs participated in the study (Table 1). Participants’ data have been used in a previous study, which focused on intrinsic striatal activity in patients with schizophrenia during psychosis and psychotic remission (Sorg et al., 2013). In particular, data from patients in psychotic remission were re-analyzed in the current study focusing on the relationship between insular dysfunction, aberrant inter-network interactions and negative symptoms in schizophrenia. All patients provided informed consent in accordance with the Human Research Committee guidelines of the Klinikum Rechts der Isar, Technische Universität München. Patients were recruited from the Department of Psychiatry, controls by word-of-mouth advertising. Participants’ examination included medical history, psychiatric interview, psychometric assessment, and blood tests for patients. Psychiatric diagnoses were based on DSM-IV (American Psychiatric Association, 2000). The Structured Clinical Interview for DSM-IV [SCID-I (Spitzer et al., 1992)] was used to assess the presence of psychiatric diagnoses. Severity of clinical symptoms was measured with the Positive and Negative Syndrome Scale (PANSS) (Kay et al., 1987) on the day of scanning. Psychiatrists Dirk Schwerthöffer and Martin Scherr, who performed clinical-psychometric assessment, have been professionally trained for SCID and PANSS-based interviews with inter-rater reliability for diagnoses and scores of more than 95%. The global level of social, occupational, and psychological functioning was measured with the Global Assessment of Functioning Scale (GAF) (Spitzer et al., 1992).

**Table 1:** **Demographic and clinical characteristics**.

Measure	SR (*n* = 12)	HC (*n* = 12)	SR vs. HC^1^	
	Mean (SD)	Mean (SD)	*T*-score	*p*-Value
Age	32.50 (10.04)	34.67 (12,25)	−0.474	0.640
Sex (m/f)	4/8	4/8		
PANSS				
Total	53.09 (14.56)	30.41 (1.44)	5.379	<0.001*
Positive	12.09 (3.75)	7.08 (0.29)	4.824	<0.001*
Negative	13.08 (5.95)	7.17 (0.58)	3.431	0.002*
General	27.36 (8.69)	16.17 (0.58)	4.458	<0.001*
GAF	59.09 (15.14)	99.17 (2.89)	−9.013	<0.001*
CPZ	207.42 (198.12)			
Duration of illness (years)	4.11 (3.29)			

^1^Two-sample *t*-test; *significant for *p* < 0.05, Bonferroni-corrected for multiple comparisons.

*SR, patients with schizophrenia during state of remission; HC, healthy control group; PANSS, Positive and Negative Syndrome Scale; GAF, Global Assessment of Functioning Scale; CPZ, chlorpromazine equivalent dose*.

All patients were diagnosed with schizophrenia and were ambulatory during state of remission at the time-point of scanning. Further inclusion criteria were age between 18 and 60 years and remission of psychotic symptoms [as indicated by significantly decreased PANSS scores compared to the admission during state of acute psychosis, see (Sorg et al., 2013) for detailed presentation of clinical characteristics at time-point of admission]. On average about 10 months after psychosis (*t*_mean_ = 306.08 days, *t*_SD_ = 278.72 days), patients approved an investigation during state of remission. Patients were free of any current or past neurological or internal systemic disorder, current or past depressive or manic episode, substance abuse (except nicotine), and cerebral pathology in MRI. The mean duration of illness was 4.11 years (SD = 3.29 years), the mean number of hospital stays was 4.00 (SD = 1.07). Four out of 12 patients were free of antipsychotic medication. All other patients received mono- or dual therapy with atypical antipsychotic medication, including Amisulpride (*n* = 1 case), Olanzapine (*n* = 1), Clozapine (*n* = 3), Quetiapine (*n* = 3), Risperidone (*n* = 2), and Aripiprazole (*n* = 1) (see Table 2 for individual medication protocols and dosage and Table 1 for mean chlorpromazine (CPZ) equivalent dose (Woods, 2003). All controls were free of any current or past psychiatric, neurological or systemic disorder or psychotropic medication.

**Table 2:** **Individual subject medication protocol and dosage**.

Participants	Scan during state of remission
1	400 mg Clozapine
2	NO medication
3	2 mg Risperidone
4	NO medication
5	12.5 mg Olanzapine
6	NO medication
7	NO medication
8	300 mg Clozapine
9	600 mg Quetiapine
10	600 mg Amisulpride, 400 mg Quetiapine
11	600 mg Quetiapine, 5 mg Risperidone
12	450 mg Clozapine, 15 mg Aripiprazole

All participants underwent 10 min of rs-fMRI with the instruction to keep their eyes closed and not to fall asleep. We verified that subjects stayed awake by interrogating via intercom immediately after the rs-fMRI scan. Before and after scanning, a medical examination of patients validated their stable condition and investigated whether they had feelings of odd situations during the scanning. No patient dropped out during the scanning session.

### MRI data acquisition

MRI was performed on a three T MR scanner (Achieva, Philips, Netherlands) using an eight-channel phased-array head coil. For co-registration and volumetric analysis, T1-weighted anatomical data were obtained by using a magnetization-prepared rapid acquisition gradient echo sequence (TE = 4 ms, TR = 9 ms, TI = 100 ms, flip angle = 5°, FoV = 240 mm^2^ × 240 mm^2^, matrix = 240 × 240, 170 slices, voxel size = 1 mm^3^ × 1 mm^3^ × 1 mm^3^). fMRI data were obtained by using a gradient echo EPI sequence (TE = 35 ms, TR = 2000 ms, flip angle = 82°, FoV = 220 mm^2^ × 220 mm^2^, matrix = 80 × 80, 32 slices, slice thickness = 4 mm, and 0 mm interslice gap; 300 volumes).

### fMRI data analysis

#### Preprocessing

For each participant, first three functional scans of fMRI were discarded due to magnetization effects. SPM8 (Wellcome Department of Cognitive Neurology, London) was used for motion correction, spatial normalization into the stereotactic space of the Montreal Neurological Institute (MNI) and spatial smoothing with an 8 mm × 8 mm × 8 mm Gaussian kernel. To control for differences in motion between groups, excessive head motion (linear shift > 3 mm across run and on a frame-to-frame basis, rotation > 1.5°) was applied as exclusion criteria (Sorg et al., 2013). None of the participants had to be excluded. Two-sample *t*-tests between patients with schizophrenia during psychotic remission (SR) and HC yielded no significant results regarding translational (SR vs. HC: *x*-axis: *T* = −0.035, *p* = 0.972; *y*-axis: *T* = 0.478, *p* = 0.639; *z*-axis: *T* = −0.082, *p* = 0.936) and rotational movements of any direction (SR vs. HC: pitch: *T* = 0.594, *p* = 0.560; roll: *T* = 1.013, *p* = 0.325; yaw: *T* = −0.107, *p* = 0.298). Signal-to-noise ratio of fMRI data was not different between patients with schizophrenia during state of remission (mean = 46.16, SD = 11.46) and HCs (mean = 45.79, SD = 11.58)(two-sample *t*-test, *p* = 0.94).

#### Independent component analysis

Following a recently proposed approach (Allen et al., 2011), preprocessed data were decomposed into 75 spatial independent components within a group-ICA framework (Calhoun et al., 2001), based on the infomax-algorithm and implemented in the GIFT-software^1^. High-model-order ICA approaches yield independent components, which are in accordance with known anatomical and functional segmentations (Damoiseaux et al., 2006; Kiviniemi et al., 2009; Smith et al., 2009; Abou-Elseoud et al., 2010; Allen et al., 2011). fMRI data were concatenated and reduced by two-step principal component analysis, followed by independent component estimation with the infomax-algorithm. We subsequently ran 20 ICA (ICASSO) to ensure stability of the estimated components. This results in a set of average group components, which are then back-reconstructed into single-subject space. Each back-reconstructed component consists of a spatial z-map reflecting component’s FC pattern across space (intra-iFC) and an associated time course reflecting component’s activity across time.

#### Selection of model-order and networks-of-interest

**Figure 1:**
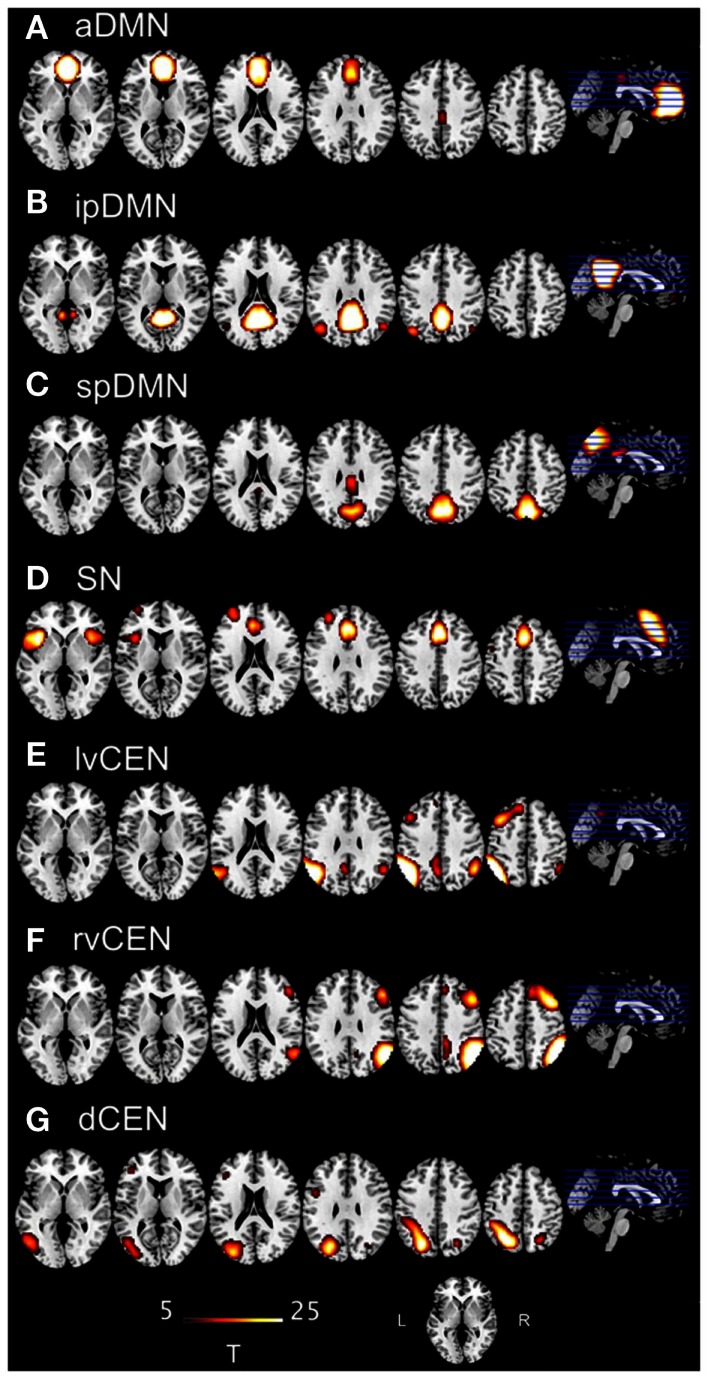
**T-maps of intrinsic connectivity networks of interest as described and provided online by Allen et al. (2011)**. Allen and colleagues used an ICA model-order of 75 to decompose rs-fMRI data of 603 subjects, obtaining 28 components. T-maps of components were provided online (http://mialab.mrn.org/data/hcp/RSN_HC_unthresholded_tmaps.nii). In the present study, we chose the T-maps of ICs representing the default mode network, salience network and central executive network, and performed multiple spatial regression analyses of our 75 independent components’ spatial maps on these templates to select the networks-of-interest in an automated and objective way. Here, provided T-maps were superimposed on a single-subject high resolution T1 image (color scale representing *t*-values from 5 to 25). **(A)** Anterior default mode network (aDMN), corresponding to Allen-IC 25. **(B)** Inferior-posterior default mode network (ipDMN), corresponding to Allen-IC 53. **(C)** Superior-posterior default-mode network, corresponding to Allen-IC 50. **(D)** Salience network (SN), corresponding to Allen-IC 55. **(E)** Left-ventral central executive network (lvCEN), corresponding to Allen-IC 34. **(F)** Right-ventral central executive network (rvCEN), corresponding to Allen-IC 60. **(G)** Dorsal central executive network (dCEN), corresponding to Allen-IC 52.

The selection of the optimal ICA model-order to analyze rs-fMRI data is still a subject of ongoing debate (see Manoliu et al., 2013 for extensive discussion). However, it has been demonstrated that a model-order around 70 components may represent an optimal level to detect between-group differences and to avoid false positive results (Abou-Elseoud et al., 2010). Bearing this in mind and exactly following a recently proposed approach of Allen et al. (2011), we decomposed our data into 75 independent components. The congruence with Allen’s approach enables greater comparability of results across studies and reduced subjective bias for ICN selection. In more detail, Allen and colleagues used an ICA model-order of 75 to decompose rs-fMRI data of 603 subjects within a group-ICA framework based on the infomax-algorithm and implemented in the GIFT-software^2^ (Calhoun et al., 2001). Authors provided T-maps of 28 components, which reflect canonical ICNs online^3^ (Allen et al., 2011). To select components, which reflect networks-of-interest, in an automated and objective way, we chose from these T-maps those representing subsystems of the SN, DMN, and CEN (7 of 28 maps, see Figure 1), and performed multiple spatial regression analyses of our 75 independent components’ spatial maps on these templates. We selected components of highest correlation coefficient with the templates, resulting in seven ICNs of interest: one component reflecting the SN, three reflecting subsystems of the DMN or CEN, respectively. In the end, this approach yielded for each subject and ICN a component’s z-map and time course, which reflect network’s coherent activity.

#### Outcome measures and statistical analysis

##### Intra-iFC

To statistically evaluate intra-iFC of selected ICs, we calculated voxel-wise one-sample *t*-tests on participants’ reconstructed spatial maps for each group, using SPM8 [*p* < 0.05, family-wise-error (FWE)-corrected for multiple comparisons]. To analyze group differences, participants’ spatial maps were entered into two-sample *t*-tests with age, sex and total GM volumes [see Voxel-based Morphometry Analysis. for detailed presentation of calculation of total GM] as covariates-of-no-interest (*p* < 0.05 FWE-corrected).

##### Inter-iFC

To statistically evaluate inter-iFC between selected ICs, subject specific ICN TCs were detrended, despiked, filtered using a fifth-order Butterworth low-pass filter with a high frequency cutoff of 0.15 Hz, and pairwise correlated by Pearson’s correlation, following the approach of Jafri et al. (2008). To assess group differences, correlation coefficients were transformed to *z*-scores using Fisher’s *z*-transformation and entered into two-sample *t*-tests with age, sex, and total GM volumes (see Voxel-Bases Morphometry Analysis. for details regarding the calculation of total GM) as covariate-of-no-interest (*p* < 0.05, Bonferroni-corrected for multiple comparisons).

##### Correlation analyses

Insular dysfunction has been suggested to be associated with various symptom dimensions, including both positive and negative symptoms in schizophrenia (Menon, 2011; Palaniyappan and Liddle, 2012). Accordingly, PANSS scores for total positive and negative symptoms were selected for further correlation analyses. To evaluate potential relationships between AI’s aberrant intra-iFC within the SN and both altered between-network interactions (inter-iFCs) and severity of symptoms in patients with schizophrenia during state of psychotic remission, we followed a recently reported analysis approach (Manoliu et al., 2013). By applying the same analysis procedures as previously reported, we were able to ensure a broad comparability between our recently reported findings in patients with schizophrenia during state of acute psychosis and the current study’s results in patients with schizophrenia during state of psychotic remission, thus providing the possibility to potentially infer on disease-state specific alterations in FC in schizophrenia. First, we calculated voxel-wise one-sample *t*-tests on patients’ reconstructed intra-iFC maps for the SN and masked the result with a mask derived from the two-sample-*t*-test contrasting patients from HCs. Subsequently, we extracted principle eigenvariates of the clusters representing intra-iFC of the left and right AI within the SN. Then we used eigenvariate-scores for partial correlation analyses of Fisher-z-transformed inter-iFC scores and PANSS scores of total positive and negative PANSS scores, respectively, including age, sex, total GM, and CPZ as covariates of no interest (see Voxel-Bases Morphometry Analysis. for detailed description of the calculation of total GM). To study the relationship between inter-iFCs and severity of symptoms in patients, we used Fisher-z-transformed inter-iFC scores for partial correlation analyses of total positive and negative PANSS scores, respectively, including age, sex, total GM, and CPZ as covariates of no interest. Results of partial correlation analyses were thresholded at *p* < 0.05, Bonferroni-corrected for multiple comparisons.

### Voxel-Based morphometry analysis

The VBM analysis followed the description provided in Manoliu et al. (2013). The FC of intrinsic brain networks depends on widespread structural integrity of polysynaptic pathways (Lu et al., 2011). Since we focus on alterations of functional interactions among networks, we included total GM scores as covariate-of-no-interest in above-mentioned FC analyses to control for this influence of structural variations. As described recently (Sorg et al., 2013), we used the VBM8 toolbox^4^ to analyze brain structure. T1-weighted images were corrected for bias-field in homogeneity, registered using linear (12-parameter affine) and non-linear transformations, and tissue-classified into GM, WM, and cerebro-spinal fluid (CSF) within the same generative model (Ashburner and Friston, 2005). The resulting GM images were modulated to account for volume changes resulting from the normalization process. Here, we only considered non-linear volume changes so that further analyses did not have to account for differences in head size. Finally images were smoothed with a Gaussian kernel of 8 mm (FWHM). For group comparisons, voxel-wise *t*-tests were performed. We applied a height threshold (voxel level) of 0.05, family-wise error (FWE) corrected. Global volumes of GM and WM were derived from the first segmentation process. Groups were compared by two-sample *t*-tests. Finally, we included total GM scores as covariate-of-no-interest in the functional analyses of ICNs.

## Results

### Intrinsic connectivity networks: Intra- and inter-iFC

**Figure 2:**
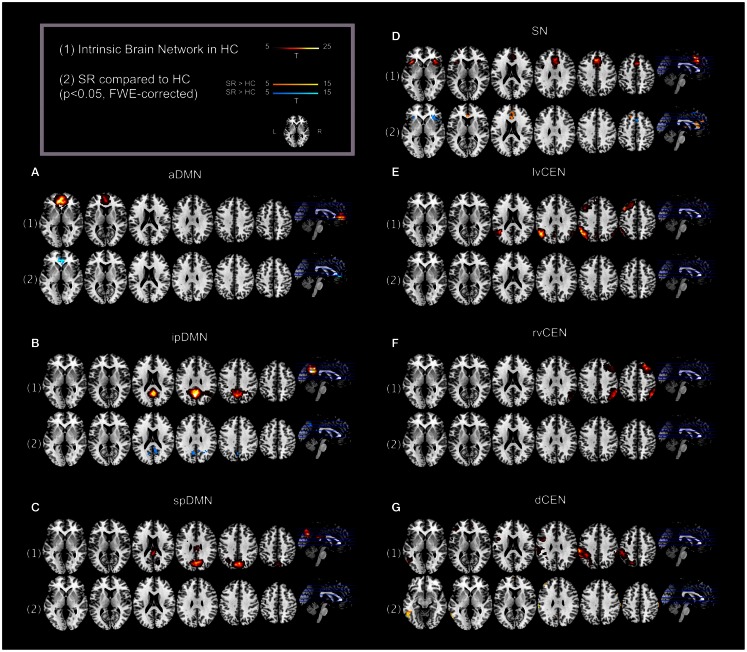
**Default mode network, salience network, and central executive network in healthy controls (HCs) and corresponding group differences for patients with schizophrenia in state of remission**. (1) Spatial maps of selected ICs representing the default mode, salience, and central executive network (DMN, SN, CEN) in HCs were entered into voxel-wise one-sample *t*-tests and thresholded at *p* < 0.05, corrected for family-wise error (FWE). Statistical parametric maps (SPMs) representing brain areas with significantly co-varying activity were superimposed on a single-subject high resolution T1 image (color scale representing *t*-values from 5 to 25; only maps of HCs are shown). (2) To analyze between-group differences, patients’ and controls’ ICs of the DMN, SN, and CEN were entered into voxel-wise two-sample-*t*-test with age, sex, and total GM volume as covariates of no interest and thresholded at *p* < 0.05, FWE-corrected. SPMs were superimposed on a single-subject high resolution T1 image (color scale representing *t*-values from 5 to 15; yellow (“hot”) color maps indicate regions displaying higher intra-iFC in SR compared to HC; blue (“cold”) color maps indicate regions displaying less intra-iFC in SR compared to HC). Results for each network of interest are presented panel-wise: **(A)** anterior default mode network (aDMN); **(B)** inferior-posterior default mode network (ipDMN); **(C)** superior-posterior default-mode network; **(D)** salience network (SN); **(E)** left-ventral central executive network (lvCEN); **(F)** right-ventral central executive network (rvCEN); **(G)** dorsal central executive network (dCEN). SR, group of patients with schizophrenia during remission; HC, healthy control group (see also Tables 3 and 4).

**Figure 3:**
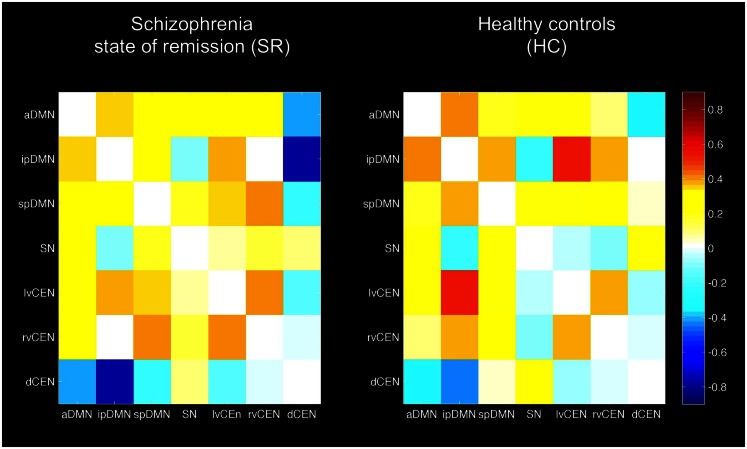
**Inter-network intrinsic functional connectivity matrix for patients with schizophrenia in state of remission and healthy controls (HCs)**. Pairwise Pearson’s correlations between time courses of the default mode, salience, and central executive network (DMN, SN, CEN) were Fisher-z-transformed, averaged across subjects for each group of patients with schizophrenia and HCs, and presented in a correlation matrix. Colors represent intensity of averaged *z*-scores. a/ip/spDMN: anterior/inferior-posterior/superior-posterior DMN; lv/rv/dCEN: left-ventral/right-ventral/dorsal CEN (see also Table 5).

In general, both intra-iFC and inter-iFC were almost perfectly in line with findings of Allen et al. (2011), indicating that the basic functional architecture of SN, DMN, and CEN was present in both groups (see Figure 1 for presentation of spatial templates, Figure 2 and Table 3 for detailed presentation of intra-iFC within ICNs of interest and Figure 3 and Table 5 for detailed presentation of inter-iFC between ICNs of interest).

**Table 3:** **Intrinsic connectivity networks in healthy controls**.

Anatomical region	L/R/Bi	cluster	*z*-Score	*p*-Value*	MNI (*x*,*y*,*z*)^1^
**(A) ANTERIOR DEFAULT MODE NETWORK (aDMN)**					
Medial prefrontal cortex	L	451	6.88	<0.001	−6, 45, 0
Medial prefrontal cortex	R	″	6.73	<0.001	6, 39, −3
**(B) INFERIOR-POSTERIOR DEFAULT MODE NETWORK (ipDMN)**					
Medial posterior parietal cortex	L	579	>8.00	<0.001	−3, −60, 30
Medial posterior parietal cortex	R	″	6.80	<0.001	6, −51, 24
Angular gyrus	R	″	6.55		48, −57, 27
**(C) SUPERIOR-POSTERIOR DEFAULT MODE NETWORK (spDMN)**					
Precuneus	Bi	344	6.53	<0.001	−9, −75, 36
Inferior parietal lobule	L	″	4.77	<0.001	−33, −37, 39
Posterior cingulate cortex	Bi	57	5.90	<0.001	−3, −36, 24
**(D) SALIENCE NETWORK (SN)**					
Anterior cingulate cortex	Bi	255	6.19	<0.001	−3, 27, 39
Insula lobe	L	77	5.91	<0.001	−39, 18, −3
Insula lobe	R	66	5.90	<0.001	36, 27, 0
**(E) LEFT-VENTRAL CENTRAL EXECUTIVE NETWORK (lvCEN)**					
Inferior parietal lobule	L	412	6.87	<0.001	−48, −63, 33
Superior frontal gyrus	L	137	6.16	<0.001	−39, 21, 51
Middle frontal gyrus	L	″	5.65	<0.001	−33, 9, 42
Inferior parietal lobule	R	42	5.00	<0.001	60, −51, 39
Precuneus	L	33	4.86	<0.001	−6, −69, 39
**(F) RIGHT-VENTRAL CENTRAL EXECUTIVE NETWORK (rvCEN)**					
Inferior parietal lobule	R	229	6.00	<0.001	42, −69, 45
Middle frontal gyrus	R	167	6.54	<0.001	30, 24, 45
Middle cingulate cortex	R	70	5.25	<0.001	9, −27, 36
Middle orbital gyrus	R	22	4.81	<0.001	30, 57, −6
**(G) DORSAL CENTRAL EXECUTIVE NETWORK (dCEN)**					
Supramarginal gyrus	L	300	6.35	<0.001	−60, −30, 39
Inferior temporal gyrus	L	24	5.96	<0.001	−51, −57, −6
Inferior frontal gyrus	L	12	5.20	<0.001	−48, 3, 33
Supramarginal gyrus	R	7	5.19	<0.001	63, −42, 30

*One-sample-*t*-test, significant for *p* < 0.05, FWE-corrected for multiple comparisons, cluster-threshold > 10 voxel. ^1^MNI, Montreal Neurological Institute; L, left hemisphere; R, right hemisphere; Bi, bilateral (see also Figure 2).

#### Intra-iFC

Automated component selection, which was based on spatial templates representing subsystems of the DMN, SN, and CEN (see Figure 1 for presentation of spatial templates), revealed seven components of interest for each individual: the SN was represented in one component. The DMN was represented in three components [anterior DMN (aDMN), inferior-posterior DMN (ipDMN), superior-posterior DMN (spDMN)]. The CEN was represented in three components [left-ventral CEN (lvCEN), right-ventral CEN (rvCEN), dorsal CEN (dCEN)]. Selected components were spatially consistent across groups and matched previous results of SN, DMN, and CEN (Allen et al., 2011) (see Figure 2; Table 3 for detailed description of intra-iFC within selected ICNs, *p* < 0.05, FWE-corrected).

#### Inter-iFC

Inter-iFC between intrinsic networks matched results of Allen et al. (2011) (see Figure 3; Table 5 for detailed description of inter-iFC between all network-pairs). Noteworthy, we found positive correlations between distinct subsystems of the DMN and CEN in both groups. Although this is inconsistent with previously described patterns of anti-correlation between these two networks (Fox and Raichle, 2007), it is well in line with recent findings using high-model-order ICA (Allen et al., 2011). Furthermore, Smith et al. (2012) identified several sub-networks within the DMN, each associated with characteristic patterns of inter-network connectivity by using high temporal resolution resting-state fMRI.

### Intra-iFC of the SN is disrupted in bilateral anterior insula in patients with schizophrenia during remission

Compared to HCs, patients demonstrated altered intra-iFC within the DMN, SN, and CEN. (Figure 2; Table 4; *p* < 0.05 FWE-corrected with age, sex, and total GM as covariates-of-no-interest). Regarding the SN, patients showed decreased intra-iFC within the bilateral AI. Furthermore, intra-iFC was increased in bilateral ACC within the SN (see Figure 2D). Regarding the DMN, patients showed decreased intra-iFC in bilateral ACC within the aDMN (see Figure 2A) and decreased intra-iFC in bilateral precuneus within the ipDMN (see Figure 2B). No between-group differences were observed within the spDMN. Regarding the CEN, patients showed increased intra-iFC in the left inferior temporal gyrus within the dCEN (see Figure 1G). No between-group differences were observed within both lvCEN and rvCEN.

**Table 4:** **Altered intra-iFC in patients with schizophrenia in state of remission compared to healthy controls**.

Anatomical Region	L/R/Bi	cluster	*z*-Score	*p*-Value*	MNI (*x*,*y*,*z*)^1^
**(A) ANTERIOR DEFAULT MODE NETWORK (aDMN)**					
(a) SR > HC					
–	–	–	–	–	–
(b) SR < HC					
Anterior cingulate cortex	Bi	179	>8.00	<0.001	9, 42, −3
**(B) INFERIOR-POSTERIOR DEFAULT MODE NETWORK (ipDMN)**					
(a) SR > HC					
–	–	–	–	–	–
(b) SR < HC					
Precuneus	R	21	5.71	<0.001	12, −60, 24
	L	23	5.37	0.001	−9, −60, 30
**(C) SUPERIOR-POSTERIOR DEFAULT MODE NETWORK (spDMN)**					
(a) SR > HC					
–	–	–	–	–	–
(b) SR < HC					
–	–	–	–	–	–
**(D) SALIENCE NETWORK (SN)**					
(a) SR > HC					
Anterior cingulate cortex	Bi	33	5.83	<0.001	0, 27, 12
(b) SR < HC					
Insula lobe	R	18	5.68	<0.001	36, 27, 0
Insula lobe	L	8	5.08	<0.001	−27, 27, 9
**(E) LEFT-VENTRAL CENTRAL EXECUTIVE NETWORK (lvCEN)**					
(a) SR > HC					
–	–	–	–	–	–
(b) SR < HC					
–	–	–	–	–	–
**(F) RIGHT-VENTRAL CENTRAL EXECUTIVE NETWORK (rvCEN)**					
(a) SR > HC					
–	–	–	–	–	–
(b) SR < HC					
–	–	–	–	–	–
**(G) DORSAL CENTRAL EXECUTIVE NETWORK (dCEN)**					
(a) SR > HC					
Inferior temporal gyurs	L	111	7.52	<0.001	−54, −52, −21
(b) SR < HC					
–	–	–	–	–	–

*Two-sample-*t*-test with age, sex, and total GM volume as covariates of no-interest, significant for *p* < 0.05, FWE-corrected for multiple comparisons. cluster-threshold > 5 voxel. ^1^MNI, Montreal Neurological institute; L, left hemisphere; R, right hemisphere, Bi, bilateral (see also Figure 2).

### Inter-iFC between SN and CEN is increased in patients with schizophrenia during remission

**Figure 4:**
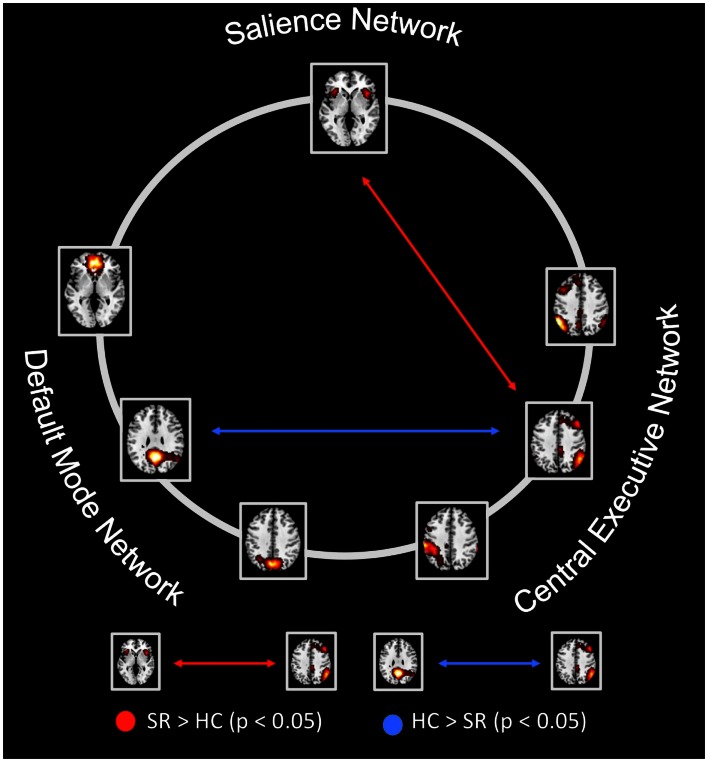
**Between-group differences of inter-network intrinsic functional connectivity**. Based on network time courses (TCs), inter-network intrinsic functional connectivity (inter-iFC) were calculated by the use of Pearson’s correlation between subject specific ICN TCs. The red arrows indicates increased inter-iFC in patients compared to healthy controls (HCs) (two-sample *t*-test, *p* < 0.05, Bonferroni-corrected for multiple comparisons); The blue arrows indicates decreased inter-iFC in patients compared to HCs (two-sample *t*-test, *p* < 0.05, Bonferroni-corrected for multiple comparisons). Spatial maps indicate the anterior/inferior-posterior/superior-posterior default mode network (a/ip/spDMN), left-ventral/right-ventral/dorsal central executive network (lv/rv/dCEN), and salience network (SN). All tests were corrected for age, sex and total GM volume. Abbreviations: SR, group of patients with schizophrenia during remission; HC, healthy control group (see also Table 5).

Compared to HCs, patients during psychotic remission showed both increased and decreased inter-iFC (Figure 4; Table 5; *p* < 0.05, corrected for age, sex, and total GM, Bonferroni-corrected for multiple comparisons). Patients showed decreased inter-iFC between ipDMN and rvCEN, suggesting a decreased FC between the DMN and CEN. Furthermore, patients showed increased inter-iFC between SN and rvCEN, indicating increased FC between the SN and CEN.

**Table 5:** **Inter-network intrinsic functional connectivity in patients with schizophrenia in state of remission and healthy controls**.

Inter-iFC	SR (*n* = 12)		HC (*n* = 12)		SR vs. HC^1^	
	Mean	SD	Mean	SD	Direction	*p*-Value
aDMN – ipDMN	0.351	0.188	0.424	0.200	HC > SR	0.266
aDMN – spDMN	0.328	0.195	0.138	0.141	SR > HC	*0.034*
aDMN – SN	0.274	0.148	0.152	0.188	SR > HC	0.141
aDMN – lvCEN	0.261	0.168	0.157	0.124	SR > HC	0.078
aDMN – rvCEN	0.312	0.131	0.105	0.173	SR > HC	*0.011*
aDMN – dCEN	−0.411	0.223	−0.318	0.121	HC > SR	0.473
ipDMN – spDMN	0.268	0.123	0.317	0.295	HC > SR	0.563
ipDMN – SN	−0.094	0.178	−0.301	0.194	SR > HC	0.052
ipDMN – lvCEN	0.387	0.195	0.545	0.195	HC > SR	0.14
ipDMN – rvCEN	0.003	0.143	0.371	0.107	*HC* > *SR*	<0.001*
ipDMN – dCEN	−0.782	0.195	−0.523	0.126	HC > SR	*0.008*
spDMN – SN	0.171	0.193	0.149	0.148	SR > HC	0.988
spDMN – lvCEN	0.343	0.176	0.162	0.267	SR > HC	0.076
spDMN – rvCEN	0.418	0.197	0.190	0.192	SR > HC	*0.021*
spDMN – dCEN	−0.222	0.216	0.032	0.229	HC > SR	0.066
SN – lvCEN	0.071	0.134	−0.140	0.240	SR > HC	0.066
SN – rvCEN	0.166	0.157	−0.177	0.237	*SR* > *HC*	0.002*
SN – dCEN	0.109	0.176	0.260	0.147	HC > SR	0.088
lvCEN – rvCEN	0.410	0.166	0.359	0.223	SR > HC	0.609
lvCEN – dCEN	−0.150	0.230	−0.119	0.159	HC > SR	0.678
rvCEN – dCEN	−0.025	0.195	−0.088	0.168	SR > HC	0.289

^1^Two-sample *t*-test, controlled for age, sex, and total GM volume. Italics indicate *p* < 0.05; *significant for *p* < 0.05, Bonferroni-corrected for multiple comparisons (*n* = 21).

*SR, group of patients with schizophrenia during remission; HC, healthy control group; inter-iFC, inter-network intrinsic functional connectivity; a/ip/spDMN: anterior/inferior-posterior/superior-posterior DMN; lv/rv/dCEN: left-ventral/right-ventral/dorsal CEN; SN: salience network (see also Figures 3 and 4)*.

### Left anterior insula’s aberrant SN connectivity is associated with altered SN-CEN interaction in patients with schizophrenia during remission

**Figure 5:**
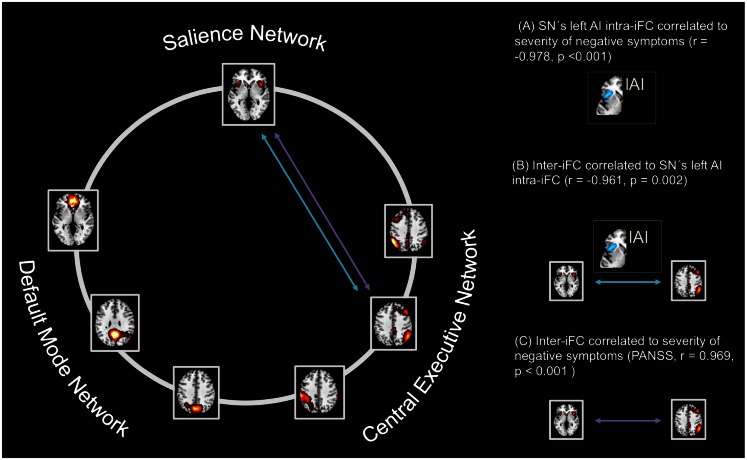
**Intra-iFC in the left anterior insula within the salience network is associated with increased SN-CEN interaction and severity of negative symptoms**. Intrinsic functional connectivity (inter-iFC) between ICNs of interest was calculated by the use of Pearson’s correlation between-networks’ time courses. **(A)** Intra-iFC in the left anterior insula within the SN (turquoise spatial map) was significantly correlated with severity of negative symptoms in patients (partial correlation, *r* = −0.978, *p* < 0.001). **(B)** Furthermore, intra-iFC in the left anterior insula within the SN was significantly correlated with the inter-iFC between SN and CEN in patients (turquoise arrow, partial correlation, *r* = −0.961, *p* = 0.002). **(C)** Finally, the inter-iFC between SN and CEN was significantly correlated with the severity of negative symptoms (purple arrow, partial correlation, *r* = 0.969, *p* < 0.001). All partial correlations were corrected for age, sex, total GM volume, and medication (CPZ). Spatial maps indicate the anterior/inferior-posterior/superior-posterior default mode network (a/ip/spDMN), left-ventral/right-ventral/dorsal central executive network (lv/rv/dCEN), and salience network (SN) (see also Tables 6– 8).

To study the influence of insular SN activity on altered inter-network connectivity in patients, we correlated eigenvariates of SN’s left and right AI group difference clusters with Fisher-z-transformed correlation coefficients of each pair of network TCs (Figure 5; Table 6, *p* < 0.05, partial correlations with age, sex, total GM, and CPZ as covariates of no-interest, Bonferroni-corrected for multiple comparisons). In patients, SN’s left AI intra-iFC correlated negatively with inter-iFC between SN and rvCEN (*r* = −0.96). There was no further significant correlation of SN’s right or left AI intra-iFC with inter-iFC scores.

**Table 6:** **Partial correlations between intra-iFC in the right/left AI within the SN and inter-iFC in patients with schizophrenia in state of remission**.

Inter-iFC	right AI		left AI	
	*r*-score	*p*-Value	*r*-score	*p*-Value
aDMN – ipDMN	0,056	0,916	−0,603	0,205
aDMN – spDMN	−0,015	0,977	0,507	0,305
aDMN – SN	−0,671	0,145	−0,009	0,987
aDMN – lvCEN	0,232	0,658	0,392	0,442
aDMN – rvCEN	0,101	0,849	0,793	0,06
aDMN – dCEN	−0,604	0,204	0,605	0,204
ipDMN – spDMN	0,306	0,555	−0,698	0,123
ipDMN – SN	−0,05	0,925	0,038	0,943
ipDMN – lvCEN	0,672	0,144	−0,132	0,803
ipDMN – rvCEN	0,819	*0,046*	−0,24	0,646
ipDMN – dCEN	−0,374	0,466	0,293	0,572
spDMN – SN	−0,034	0,949	0,84	*0,036*
spDMN – lvCEN	0,049	0,926	−0,236	0,653
spDMN – rvCEN	−0,407	0,423	−0,597	0,21
spDMN – dCEN	−0,28	0,591	0,493	0,321
SN – lvCEN	−0,602	0,206	0,238	0,65
SN – rvCEN	0,207	0,695	−0,961	0,002*
SN – dCEN	−0,528	0,281	0,956	*0,003*
lvCEN – rvCEN	0,356	0,488	−0,491	0,322
lvCEN – dCEN	−0,779	0,068	−0,037	0,945
rvCEN – dCEN	−0,362	0,481	0,319	0,538

Italics indicate *p* < 0.05; *significant for *p* < 0.05, Bonferroni-corrected for multiple comparisons (*n* = 21). Partial correlation, corrected for age, sex, total GM volume and chlorpromazine equivalent dose (CPZ).

*a/ip/spDMN: anterior/inferior-posterior/superior-posterior DMN; lv/rv/dCEN: left-ventral/right-ventral/dorsal CEN; SN: salience network; AI: anterior insula (see also Figure 5)*.

### Left anterior insula’s aberrant SN connectivity is associated with severity of negative symptoms in patients with schizophrenia during remission

To study the influence of insular SN activity on the severity of positive and negative symptoms in patients, we correlated eigenvariates of SN’s left and right AI group difference clusters with PANSS scores for total positive symptoms and total negative symptoms, respectively (Figure 5; Table 7; *p* < 0.05, partial correlations with age, sex, total GM, and CPZ as covariates of no-interest, Bonferroni-corrected for multiple comparisons). In patients, SN’s left AI’s intra-iFC correlated negatively with the severity of total negative symptoms (*r* = −0.97) but not with the severity of total positive symptoms. Furthermore, SNs right AI’s intra-iFC correlated positively with the severity of total positive symptoms (*r* = 0.886). However, this result was not significant when corrected for multiple comparisons. There was no further significant correlation of SN’s right or left AI intra-iFC with behavioral scores.

**Table 7:** **Partial correlations between intra-iFC in the right/left AI within the SN and severity of positive and negative symptoms in patients with schizophrenia in state of remission**.

PANSS scores	right AI		left AI	
	*r*-Score	*p*-Value	*r*-Score	*p*-Value
**(A) TOTAL SCORES**				
Total positive symptoms	0,886	*0,019*	−0,553	0,255
Total negative symptoms	0,141	0,789	−0,978	0,001*

Italics indicate *p* < 0.05; *significant for *p* < 0.05, Bonferroni-corrected for multiple comparisons (*n* = 4). Partial correlation, corrected for age, sex, total GM volume and chlorpromazine equivalent dose (CPZ).

*AI: anterior Insula; PANSS: Positive and Negative Syndrome Scale (see also Figure 5)*.

### Impaired SN-CEN interaction is selectively associated with severity of negative symptoms

To study the relationship of between-network interactions with severity of positive and negative symptoms, we correlated inter-iFC scores with PANSS scores for both total positive symptoms and total negative symptoms, respectively (Figure 5; Table 8; *p* < 0.05, partial correlations with age, sex, total GM, and CPZ as covariates of no-interest, Bonferroni-corrected for multiple comparisons). Inter-iFC between SN and rvCEN correlated positively with the severity of total negative symptoms (*r* = 0.969) but not with the severity of positive symptoms. There was no further significant correlation of inter-iFC across network-pairs with behavioral scores.

**Table 8:** **Partial correlations between inter-iFC and severity of positive and negative symptoms in patients with schizophrenia in state of remission**.

Inter-iFC	Total Positive Symptoms		Total Negative Symptoms	
	*r*-score	*p*-Value	*r*-score	*p*-Value
aDMN – ipDMN	0.443	0.379	0.595	0.213
aDMN – spDMN	−0.254	0.627	−0.574	0.233
aDMN – SN	−0.717	0.109	0.135	0.799
aDMN – lvCEN	−0.152	0.774	−0.402	0.429
aDMN – rvCEN	−0.243	0.643	−0.869	*0.025*
aDMN – dCEN	−0.764	0.077	−0.463	0.355
ipDMN – spDMN	0.361	0.482	0.597	0.211
ipDMN – SN	−0.184	0.727	−0.016	0.977
ipDMN – lvCEN	0.807	0.052	0.071	0.893
ipDMN – rvCEN	0.844	*0.034*	0.105	0.844
ipDMN – dCEN	−0.166	0.753	−0.129	0.808
spDMN – SN	−0.256	0.624	−0.865	0.026
spDMN – lvCEN	−0.03	0.955	0.188	0.722
spDMN – rvCEN	−0.115	0.828	0.643	0.169
spDMN – dCEN	−0.327	0.526	−0.362	0.481
SN – lvCEN	−0.615	0.194	−0.073	0.89
SN – rvCEN	0.555	0.253	0.969	<0.001*
SN – dCEN	−0.686	0.133	−0.9	*0.014*
lvCEN – rvCEN	0.396	0.437	0.486	0.329
lvCEN – dCEN	−0.728	0.101	0.179	0.735
rvCEN – dCEN	−0.509	0.302	−0.228	0.663

Italics indicate *p* <0.05; *significant for *p* < 0.05, Bonferroni-corrected for multiple comparisons (*n* = 21). Partial correlation, corrected for age, sex, total GM volume and chlorpromazine equivalent dose (CPZ).

*a/ip/spDMN: anterior/inferior-posterior/superior-posterior DMN; lv/rv/dCEN: left-ventral/right-ventral/dorsal CEN; SN: salience network (see also Figure 5)*.

### Alterations in intra-iFC and inter-iFC are not explained by brain structure or medication

Regarding potential alterations in brain structure, voxel-wise tests yielded no regional GM or WM differences between groups. Although slightly decreased, total GM was not significantly changed in patients (*T* = −0.16, *p* = 0.98). Regarding potential effects of medication, we correlated CPZ with both intra-iFC of each ICN and inter-iFC for each pair of ICNs. CPZ showed no significant effect on both intra- (*p* < 0.05, FWE-corrected) and inter-iFC (*p* < 0.05, corrected for multiple comparisons), respectively. In addition, we included total GM and CPZ-scores as covariate-of-no-interest in the functional analyses of ICNs to account for these measures as potential confounders.

## Discussion

To test our hypothesis that insular dysfunction, altered between-network interactions, and negative symptoms are related in schizophrenia during psychotic remission, we investigated the intrinsic FC within- and between the SN, DMN, and CEN in patients with schizophrenia during psychotic remission and HCs. We found decreased intra-iFC in the left anterior insular cortex within the SN as well as increased inter-iFC between the SN and CEN. Furthermore, these alterations were related to each other and associated with the severity of negative symptoms. In addition, we found a strong trend for the association between decreased intra-iFC within the right AI and patients’ positive symptoms, corresponding to our previous finding in psychotic patients. This result extends our knowledge about insular dysfunction in schizophrenia by demonstrating a link between left anterior insular dysfunction, altered inter-network connectivity and negative symptoms, which is present during psychotic remission. Together with our previous result of impaired right anterior insula dysfunction in psychosis, data suggest specific SN/DMN/CEN reorganization in schizophrenia with distinct insular pathways for distinct symptom dimensions.

### The salience network in psychotic remission

#### The link between insular dysfunction within the SN, aberrant inter-network connectivity, and severity of symptoms in psychotic remission

In accordance to our hypothesis (Menon, 2011; Palaniyappan and Liddle, 2012), we found both altered intra-iFC in the left AI within the SN and altered inter-iFC between the SN and CEN. We demonstrated that both findings are related to each other (Figure 5; Table 6) and to the severity of negative symptoms in patients (Figure 5; Tables 7 and 8), indicating an association between insular dysfunction and aberrant inter-network connectivity in patients with schizophrenia during psychotic remission. Noteworthy, the right anterior insula, which showed also decreased intra-iFC within the SN, yielded a trend for a correlation with the severity of positive symptoms (*r* = 0.89, *p* = 0.02). Although this result is well in line with previous findings (Palaniyappan et al., 2012; Manoliu et al., 2013), and current models of insular dysfunction in psychosis (Menon, 2011; Palaniyappan and Liddle, 2012), it did not survive correction for multiple comparisons. This missing significance might be explained by small statistical power due to the limited size of our patient sample and low levels of variance of positive symptoms in patients (see also Limitations). All tests were performed including age, sex, total GM and CPZ as covariates-of-no-interest. Therefore, it is unlikely that present results are explained by these factors. Taken together, data demonstrate that dysfunction of the left AI within the SN in schizophrenia is present during psychotic remission and related to both altered inter-network connectivity and severity of patients’ negative symptoms.

These results are in line within the suggested disruption of the AI’s control function for between-network interactions in schizophrenia, which may persist even during psychotic remission and may be related to distinct symptom dimensions (Palaniyappan and Liddle, 2012). Several findings support this idea: Firstly, the AI has been demonstrated to play a critical role regarding the modulation of between-network interactions (Sridharan et al., 2008; Menon and Uddin, 2010). Secondly, alterations within the AI such as structural reorganization or aberrant reward-related activity have been shown in patients with schizophrenia during psychotic remission and to be linked with negative symptoms (Palaniyappan et al., 2011; Gradin et al., 2013). Thirdly, the current findings correspond with previous findings demonstrating that an impaired dependence of aberrant between-network interactions on right insular dysfunction is related with positive symptoms (Manoliu et al., 2013). Fourthly, recently formulated models providing a link between aberrant engagement and disengagement of large-scale intrinsic connectivity networks and psychopathology suggest an impaired control function of the AI in patients with schizophrenia, giving rise to both positive and negative symptoms (Menon, 2011; Palaniyappan and Liddle, 2012). Therefore, the present results suggest that anterior insular dysfunction may contribute to symptoms of schizophrenia via aberrant inter-network interaction.

Our findings suggest an asymmetric involvement of the AI in patients with schizophrenia as a function of state of disease. While the intra-iFC within the left AI was associated with both altered interactions between SN and CEN and severity of negative symptoms in patients during state of remission (Figure 5, Tables 6 and 7), the intra-iFC within the right AI was associated with both altered interactions between the DMN and CEN and severity of positive symptoms in patients during state of psychosis [(Manoliu et al., 2013), see also Table 7]. This observation corresponds to the asymmetric representation of body-related interoceptive information in the AI, which has been suggested to originate from the asymmetry of the peripheral autonomic nervous system; the left AI is more associated with the parasympathetic system, the right AI more with the sympathetic system (Craig, 2002). It has been suggested that this asymmetric autonomous representation in the AI might underlie asymmetric representations of emotions and interoceptive awareness (Craig, 2009). For example, the right AI is more involved in “sympathetic” emotions induced by stimuli that increase arousal and energy costs of behavioral responses such as pain or aversive pictures (Craig, 2009), while the left AI is more related to positive emotions such as maternal and romantic love, joy, or positive reactions induced by pleasant stimuli, and relaxation (Craig, 2009). Considering the left AI’s role in processing positive and affiliative emotional feelings (Craig, 2009), deficits within the left AI might be associated with negative symptoms in schizophrenia (Palaniyappan and Liddle, 2012). For example, negative symptoms such as anhedonia and diminished social interactions might be associated with anomalies within the left anterior insula via impaired responses on pleasant stimuli. Accordingly, structural deficits within the anterior insular cortex have been demonstrated to be highly related to the severity of negative symptoms in patients with schizophrenia (Koutsouleris et al., 2008) while Horn et al. (2010) demonstrated a relationship between altered connectivity between AI and ACC and the severity of affective symptoms in patients with major depressive disorder. Bearing these findings in mind, our results might represent a first hint toward a relationship between asymmetric interoceptive-emotional representation in the left and right AI and the AI’s asymmetric association with positive and negative symptoms in schizophrenia as a function of state of disease. However, it is to note that we did not explicitly test for asymmetry in the present study. Future studies investigating the potential link between aberrant intrinsic FC within the left and right AI and positive and negative symptoms in patients with schizophrenia during both psychosis and psychotic remission are necessary to improve our understanding of the left and right AI’s relevance for distinct symptom dimensions in schizophrenia.

#### Further observations

In the following we want to make three further comments that may help to better evaluate and contextualize our findings centered on the SN.

##### Potential inconsistency with previous findings

In contrast to the current study, Woodward et al. (2011) found no significant findings regarding the intra-iFC of the SN in patients with schizophrenia. More specifically, the authors observed a non-significant trend to decreased network connectivity within the SN by applying a seed-based region-of-interest correlation analysis to calculate SN’s iFC in a combined group of patients with schizophrenia and schizoaffective disorder. According to the evaluation of the reported coordinates for the seeds using the “SPM Anatomy toolbox” (Eickhoff et al., 2005), the seeds were placed in the left and right inferior frontal gyrus pars orbitalis, near to the AI. In contrast, we investigated selectively patients with schizophrenia, once during state of acute psychosis in a previous study (Manoliu et al., 2013) and once during state of psychotic remission in the current study by the use of an ICA-approach. Our analyses yielded consistently aberrant intra-iFC in both AI and ACC within the SN in patients with schizophrenia during both state of acute psychosis and state of remission. Although Woodward and colleagues also found a trend for reduced intra-iFC within the SN, these contradictory results might be explained by different methodological approaches, including the exact position of the seed as reported in Woodward et al. (2011) and, maybe more important, by the highly different composition of the patient samples.

##### Findings beyond altered interactions within and between the SN, DMN, and CEN in schizophrenia

Although increasing evidence for functional (White et al., 2010; Gradin et al., 2013; Manoliu et al., 2013) and structural alterations (Palaniyappan et al., 2012) within the salience network of patients with schizophrenia points at the important role of aberrant SN-centered triple network interactions in schizophrenia (Menon, 2011), it is unclear whether and how findings beyond the SN, DMN, and CEN link with such altered triple network properties. For example Williamson and colleagues argue that models considering only the connectivity within and between SN, DMN, and CEN miss to account for both known alterations within auditory networks in patients with schizophrenia and differences between schizophrenia and other neuropsychiatric disorders demonstrating also altered FC within the SN (Williamson and Allman, 2012). Furthermore, it is unknown how aberrant iFC within subcortical regions such as the striatum (Sorg et al., 2013) or neurochemical anomalies such as increased dopaminergic activity during psychosis (Howes et al., 2009; Howes et al., 2012) are related with altered interactions between these three networks. Future studies are necessary to investigate the relationship between altered connectivity within and between the SN, DMN, and CEN and anomalies the triple network model (Menon, 2011) does not account for. It is an important research question whether the integrative potential of the SN-centered triple network model can be extended to allow also for further reported findings such as alterations in auditory networks, subcortical structures, and neurochemical activity.

##### Proximal and motivational salience in schizophrenia

Current results are well in line with the aberrant proximal salience model of Palaniyappan and Liddle (2012). Proximal salience refers to a momentary interoceptive state, which results from the evaluation of internal/external stimuli; it is represented by the SN activity particularly the AI, and it modulates both subsequent choices of actions/cognitions and learning processes to optimize evaluation; this modulation includes the control of DMN/CEN interactions via AI signals. Palaniyappan and colleagues suggest that AI/SN-related proximal salience is impaired in patients with schizophrenia contributing to distinct symptom dimensions. It is obvious that our findings support this model. Noteworthy, the concept of proximal salience is distinct from the more popular idea of motivational salience and its relevance for psychotic symptoms via aberrant prediction error processing (Kapur, 2003). Motivational salience refers to the assignment of motivational value to an external/internal stimulus after the stimulus has been evaluated; this process depends on the reward prediction error, which in turn is associated with aberrant dopamine activity in the striatum of psychotic patients. This model is in line with broader models of schizophrenia, which suggest aberrant prediction error processing as critical element underlying patients’ positive symptoms, taking the huge body of evidence for aberrant striatal dopamine in psychotic patients into account (Murray et al., 2008; Fletcher and Frith, 2009). As mentioned above, it seems to be important to study how these two concepts of aberrant salience link in schizophrenia, i.e., in terms of our finding: how do aberrant AI interactions relate with aberrant striatal prediction error activity?

### DMN/CEN interactions in psychotic remission

#### Intra-iFC within the DMN in psychotic remission

Compared to HCs, patients showed decreased intra-iFC in both ACC and PCC within the DMN, while inter-iFC between DMN’s subsystems was not altered. Although alterations in FC within the DMN in patients with schizophrenia are frequently reported during both task (Garrity et al., 2007) and rest (Whitfield-Gabrieli and Ford, 2012), the nature of this alterations remains still unclear. For instance, recent fMRI studies investigating the FC within the DMN demonstrated both decreased (Camchong et al., 2011) and increased (Whitfield-Gabrieli et al., 2009) intra-iFC in patients with schizophrenia. Among other things, inhomogeneous patient samples, often including patients during both state of psychosis and state of remission, and the application of not-standardized methodological approaches might account for this contradictory results (Whitfield-Gabrieli and Ford, 2012).

In the present study, we adopted a recently proposed pipeline for ICA of resting-state fMRI data (Allen et al., 2011) to obtain canonical ICNs in a robust and reproducible way, thus allowing for better comparability with studies using the same approach. Previously, we found decreased intra-iFC within as well as increased inter-iFC between distinct subsystems of the DMN in patients with schizophrenia during psychosis using the same methodological approach (Manoliu et al., 2013). Furthermore, the absence of increased FC between distinct subsystems of the DMN in psychotic remission is well in line with current literature, suggesting a relationship between increased FC within the DMN, severity of positive symptoms and psychosis (Garrity et al., 2007). Taken these findings together, our data suggest an aberrant intrinsic FC within the DMN as a function of state of disease.

#### Intra-iFC within the CEN in psychotic remission

Compared to HCs, patients showed increased intra-iFC in the left inferior temporal gyrus, while inter-iFC between CEN’s subsystems was not altered. Heterogeneous alterations within the CEN have been reported in schizophrenia, including both increased and decreased intra-iFC within the CEN during rest (Woodward et al., 2011). Following the above-mentioned argument for the DMN, inconsistent findings of aberrant intra-iFC within the CEN in schizophrenia might be due to both heterogeneous patient samples and distinct methodological approaches (Whitfield-Gabrieli and Ford, 2012). Previously, we found both increased and reduced intra-iFC within the CEN in psychotic patients (Manoliu et al., 2013). Due to the identical methodological approaches applied in the previous and current study, the present data suggest that the aberrant intrinsic FC within the CEN may depend on the state of disease.

#### Inter-iFC between DMN and CEN in psychotic remission

Compared to HCs, patients showed decreased inter-iFC between ipDMN and rvCEN, suggesting an aberrant inter-network connectivity between DMN and CEN. It has been suggested that schizophrenia is characterized by a disrupted relationship between the task-negative DMN and task-positive CEN (Williamson, 2007), which might underlie both positive and negative symptoms (Menon, 2011; Palaniyappan and Liddle, 2012). In particular, aberrant recruitment of anti-correlated networks has been demonstrated in schizophrenia (Hasenkamp et al., 2011). Furthermore, we demonstrated aberrant connectivity within DMN and CEN in patients with schizophrenia during acute psychosis (Manoliu et al., 2013). Our current result extends this finding by demonstrating that impaired between-network interactions in schizophrenia are also present during psychotic remission.

### Limitations

We acknowledge several limitations, which have to be considered in the present study. Firstly, antipsychotic drugs have been shown to have an impact on FC in patients with schizophrenia (Sambataro et al., 2010). However, only 4 out of 12 patients were free of antipsychotic medication, while all other patients received mono- or dual therapy with atypical antipsychotic medication. To account for this potential confounder, the total current CPZ equivalent dose was calculated and entered as covariate of no interest in all corresponding analyses. Furthermore, CPZ-scores had no significant effect on both intra-iFC and inter-iFC. Nevertheless, CPZ was entered as a linear covariate, thus not ruling out non-linear effects of antipsychotic medication. Moreover, the possible effects of different antipsychotic drugs on BOLD activity are currently not completely understood. In addition to these observations, antipsychotic drugs have in most cases an effect on positive symptoms but not on negative symptoms, potentially being reflected in a higher standard deviation of negative symptoms compared to positive symptoms in our patient sample and thus complicating the investigation of the relation between SN dysfunction and psychotic symptoms. Therefore, the present results should be interpreted with care until replicated in an unmedicated patient sample.

Secondly, limitations of the ICA have to be taken into consideration, including the arbitrary model-order selection and subjective bias in selection of the components of interest (Cole et al., 2010). Bearing this in mind, we adopted a recently proposed analysis pipeline (Allen et al., 2011) to provide a better comparability with current and future studies using the same approach. A detailed discussion of this methodological limitation can be found in Manoliu et al. (2013). Finally, only 12 patients with schizophrenia during state of remission were included in this study. It has been shown that analyses of rather small patient samples can yield very robust and interpretable results (Dovern et al., 2012; Sorg et al., 2013). However, small study samples increase the risk of obtaining false-negative statistical results, possibly explaining our negative finding regarding a relationship between intra-iFC within the right AI and the severity of positive symptoms. Therefore, a replication of our results in a larger patient sample might contribute to our current understanding of insular dysfunction in schizophrenia.

### Conclusion

Results provide evidence that left anterior insular dysfunction within the SN is selectively associated with both aberrant between-network interactions and severity of negative symptoms in patients with schizophrenia during psychotic remission. Together with correspondent findings concerning the right anterior insula in patients during psychosis, these findings suggest that the relationship between insular dysfunction and altered between-network interactions is a characteristic feature of schizophrenia, with possibly distinct insular pathways for distinct symptom dimensions.

## Conflict of Interest Statement

The authors declare that the research was conducted in the absence of any commercial or financial relationships that could be construed as a potential conflict of interest.

## Footnotes

^1^http://icatb.sourceforge.net

^2^http://icatb.sourceforge.net

^3^http://mialab.mrn.org/data/hcp/RSN_HC_unthresholded_tmaps.nii

^4^http://dbm.neuro.uni-jena.de/vbm.html
